# Cold-driven biphasic vascular healing in elderly patients: 4D optical coherence tomography stratification of major adverse cardiovascular event risk based on age-environment interactions

**DOI:** 10.3389/fcvm.2025.1663394

**Published:** 2025-11-13

**Authors:** Linxing Feng, Huaxing Liu, Zhiyong Li, Lei Xing

**Affiliations:** 1 Department of Cardiology, The Second Affiliated Hospital of Harbin Medical University, Harbin, China; 2 Key Laboratory of Myocardial Ischemia, Ministry of Education, Harbin Medical University, Harbin, China

**Keywords:** neointimal hyperplasia, drug-eluting stents, optical coherence tomography, aging, cold climate, China

## Abstract

**Background:**

Biological aging and prolonged cold exposure each impair vascular healing after implantation of a drug-eluting stent. However, their combined effect—particularly in older adults living in cold climates—remains poorly understood.

**Objective:**

To evaluate the joint impact of aging and cold exposure on vascular healing and their association with major adverse cardiovascular events (MACEs) after sirolimus-eluting stent implantation.

**Methods:**

In this retrospective cohort study, 119 patients were stratified into three age groups (≤55, 56–65, and >65 years). Vascular healing was assessed using serial optical coherence tomography (OCT) at 6 and 12 months, with a focus on strut coverage, neointimal hyperplasia (NIH), and spatial heterogeneity. Cold exposure was quantified with the validated Cold Exposure Diary Questionnaire and corroborated by regional meteorological data. The primary end point was the incidence of MACEs at 12 months.

**Results:**

OCT showed delayed endothelialization at 6 months in patients above 65 years compared to younger cohorts (uncovered struts, 11.7% vs. 6.1%; *P* < 0.001), along with accelerated late-phase NIH progression (3.33 vs. 1.67 μm/month; *P* < 0.001). Prolonged cold exposure (>12 h/day) was independently associated with greater neointimal heterogeneity (*P* = 0.003) and a higher risk of MACEs (hazard ratio, 3.42; 95% CI, 1.65–7.11). A 4D Risk Score, combining OCT-derived healing metrics and cold-exposure data, predicted MACEs; however, external validation is required.

**Conclusions:**

In older patients, the interaction between aging and prolonged cold exposure results in biphasic vascular healing, characterized by early delayed endothelialization followed by excessive neointimal proliferation. The proposed 4-D Risk Score may facilitate individualized risk stratification after percutaneous coronary intervention and warrants prospective validation.

## 1 Introduction

Drug-eluting stents (DESs) have markedly reduced restenosis and target-lesion revascularization compared with bare-metal stents. Nevertheless, late-phase cardiovascular complications, such as stent thrombosis and recurrent myocardial infarction, remain common, particularly in older adults (1). A principal contributor to these events is delayed vascular healing, typified by incomplete endothelialization, uncovered struts, and neointimal heterogeneity (2). This impairment is accentuated in older individuals because of cellular senescence and age-related vascular dysfunction. Optical coherence tomography (OCT), a high-resolution intravascular imaging modality, permits precise evaluation of strut coverage, neointimal thickness, and tissue composition. Serial OCT examinations are therefore invaluable for tracking vascular healing after DES implantation, especially for assessing endothelial recovery and neointimal maturation (3). Nonetheless, the temporal dynamics of vascular healing remain incompletely understood. In particular, biphasic neointimal proliferation—an early plateau followed by delayed regrowth—has not been systematically characterized in high-risk populations. Environmental stressors, such as cold exposure, may further modulate these healing trajectories by interacting synergistically with aging, thereby aggravating endothelial dysfunction and hindering vascular repair. As OCT-derived healing metrics increasingly guide decisions on the duration of dual antiplatelet therapy, there is growing support for a shift from fixed-duration regimens to individualized, image-guided strategies (4).

Mounting evidence highlights considerable inter-individual variability in vascular healing, with older individuals more frequently displaying delayed re-endothelialization and increased neointimal heterogeneity—features closely linked to late-phase complications after stenting (5). While patient and device characteristics are well-known contributors, emerging environmental factors—particularly chronic cold exposure—are gaining attention as potential amplifiers of impaired vascular repair. Cold-induced sympathetic activation and vasoconstriction not only impair endothelial function but also increase circulating endothelin-1, a potent vasoconstrictor implicated in vascular dysfunction (6). This cascade may further exacerbate age-related endothelial fragility. Epidemiological data from 27 countries confirm a strong association between ambient cold exposure and cardiovascular mortality (7), and a recent systematic review links chronic low-temperature exposure to increased cardiopulmonary morbidity (8). Despite these associations, the effect of cold exposure on vascular-healing trajectories after DES implantation remains uninvestigated. Moreover, no previous study has integrated objective environmental-exposure metrics with longitudinal OCT data in elderly patients undergoing percutaneous coronary intervention (PCI).

The combined effects of aging and cold exposure on vascular health remain poorly understood. Most studies have relied on short-term animal models or retrospective observational data, which do not capture the long-term, synergistic influence of aging and cold exposure on vascular repair. To address this gap, we aimed to determine how aging and cold exposure jointly affect vascular healing in elderly patients after DES implantation and to evaluate their association with major adverse cardiovascular events (MACEs). We hypothesized that cold exposure amplifies age-related delays in vascular healing, thereby increasing the risk of MACEs. By integrating Cold Exposure Diary Questionnaire (CEDQ) data with serial OCT-derived healing metrics, we developed a composite model for predicting MACEs. To our knowledge, this is the first study to combine objective cold-exposure data with OCT assessments of vascular repair in elderly patients, offering novel insights into the implications of aging and cold exposure on vascular healing and the risk of MACEs.

## 2 Methods

### 2.1 Patient population

**Figure 1:**
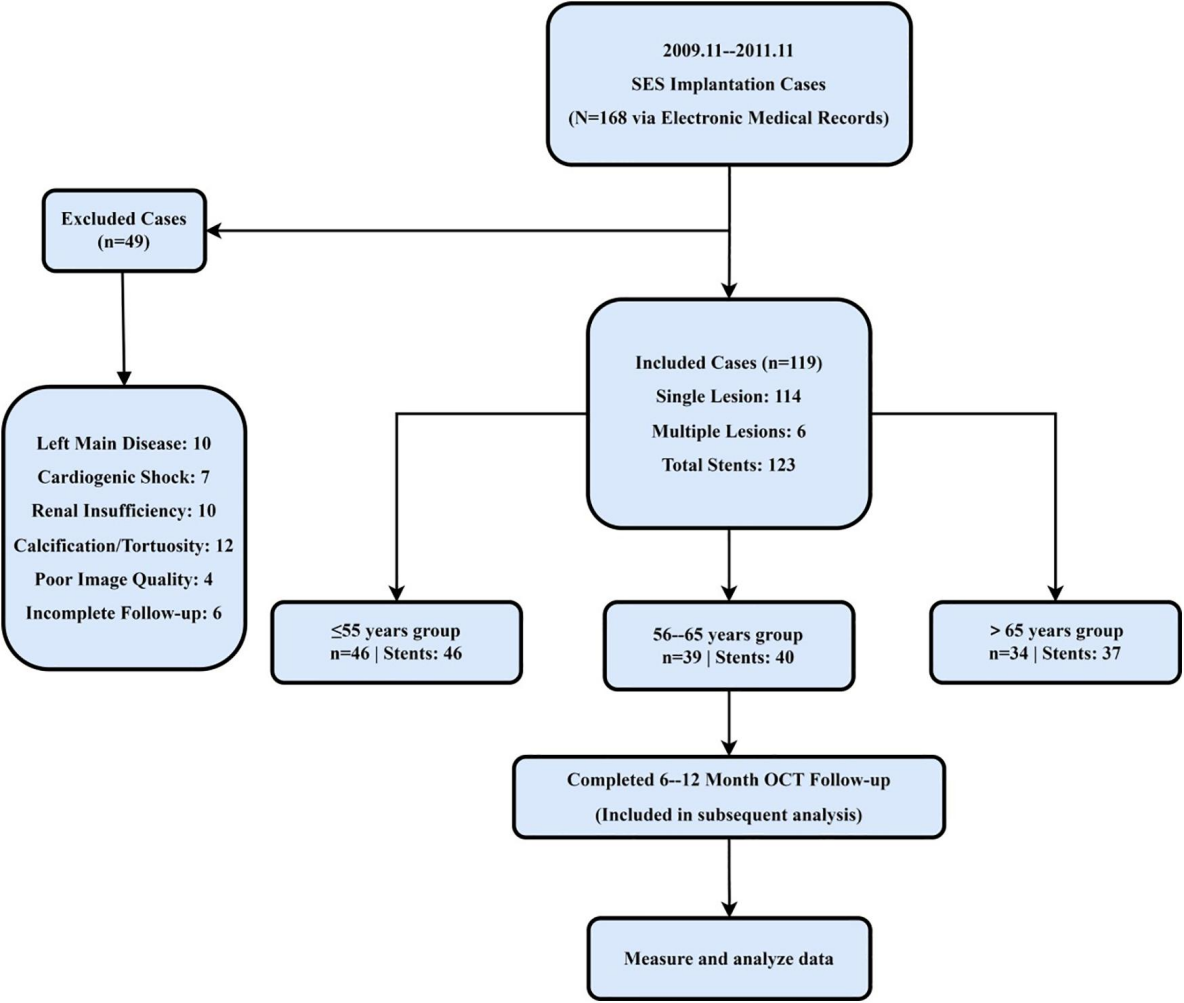
Study flow diagram and age-based stratification. A total of 119 patients undergoing DES implantation in Northeast China were screened. After excluding those with left main coronary artery disease, severe calcification, renal dysfunction, or incomplete follow-up, 119 patients were included and stratified into three age groups (≤55, 56–65, >65 years) for longitudinal OCT assessment.

This retrospective cohort study enrolled 119 consecutive patients who underwent elective or urgent PCI with sirolimus-eluting stent (SES) implantation between November 2019 and November 2021 in three north-eastern Chinese provinces—Heilongjiang, Jilin, and Liaoning. All patients had a single, *de novo* coronary lesion; in five cases, overlapping stents were placed within the same target segment. Exclusion criteria were left-main coronary artery disease, symptomatic heart failure (New York Heart Association Class III–IV), renal dysfunction (serum creatinine >1.8 mg/dl), and significant vessel tortuosity or calcification, defined as a calcium arc ≥180° or an intravascular ultrasound calcium score ≥3. Eligible patients were stratified into three age groups on the basis of previously reported age-related differences in neointimal healing and strut-coverage patterns (9): ≤55 years (*n* = 46), 56–65 years (*n* = 39), and > 65 years (*n* = 34). The patient screening and exclusion process is summarized in Figure 1. The study was approved by the Ethics Committee of Harbin Medical University, which granted a waiver of informed consent because only anonymized retrospective data were analyzed.

To ensure adequate statistical power for detecting intergroup differences in MACEs, the minimum required sample size was calculated using the following formula for comparing proportions: $\;n=\frac{Z_{\alpha /2}^{2}\cdot p(1-p)}{\delta^{2}}$, where Z*_α_*_/2_ = 1.96 (two-tailed *α* = 0.05), *p* = 0.20 (anticipated MACE incidence), and *δ* = 0.10 (margin of error). The calculated minimum sample size was 62. After accounting for potential data loss and subgroup analyses, a conservative threshold of 102 participants was set and ultimately exceeded by the final cohort of 119 patients.

### 2.2 Quantitative coronary vessel analysis

Coronary vessel analysis was performed by two independent analysts using QAngio XA (version 7.3). Target segments encompassed the stented regions and the 5-mm proximal and distal margins. Key parameters, including reference vessel diameter, minimum lumen diameter, percent diameter stenosis, and late lumen loss, were measured according to the standard protocol (10). Inter-reader discrepancies greater than 5% were resolved by consensus.

### 2.3 OCT image acquisition and analysis

OCT imaging was performed using the LightLab system (version 5.2) with a Dragonfly catheter. The system operated at a center wavelength of 1,310 nm, with an axial resolution of 15 μm and a lateral resolution of 50 μm. Pullback images were acquired at 1-mm intervals along each stented segment, excluding side branches ≥2 mm in diameter, at a pullback speed of 20 mm/s and a frame rate of 100 fps. Saline was used as the flush medium at an injection rate of 4 ml/s. Balloon occlusion was not applied during image acquisition. Image analysis was conducted independently by two reviewers following the ESC 2024 Consensus Guidelines (10). The following definitions and quantitative parameters were applied:

#### 2.3.1 Strut coverage

A strut was classified as uncovered when neointimal coverage was <40 μm at any follow-up time point, consistent with prior histopathological validation (11). Complete endothelial coverage was defined as a neointimal thickness ≥40 μm observed across three consecutive 1-mm cross-sections. Discrepancies between analysts were adjudicated by a third reviewer, resulting in excellent inter-observer agreement (Cohen's *κ* = 0.85, *P* < 0.001) (12).

#### 2.3.2 Neointimal heterogeneity

To evaluate age-related differences in neointimal healing, the interquartile range (IQR) of NIH thickness was calculated for each age group (≤55, 56–65, and >65 years). In the elderly group (>65 years), high-risk heterogeneity was defined as an IQR exceeding the 95th percentile of the cohort-wide NIH thickness distribution (13). Because anatomical and healing responses vary by lesion location, the distribution of treated vessels was documented as follows: left anterior descending (47.2%), left circumflex artery (25.2%), and right coronary artery (27.6%). Lesion location was therefore entered as a covariate in multivariable models to limit confounding. Sensitivity analyses confined to LAD lesions were also performed to test the robustness of spatial-heterogeneity findings. All OCT measurements were obtained using the LightLab system (version 5.2), which showed a strong correlation with histological benchmarks (r = 0.91). Pullback images were acquired at 1-mm intervals along each stented segment, excluding side branches ≥2 mm in diameter. Each stent was evaluated in an average of 15 ± 3 cross-sections.

#### 2.3.3 Sampling and analysis

Each frame from the pullback was analyzed, with no frames excluded from analysis. Lumen and stent contours were segmented using a semi-automated approach, with manual adjustments when necessary. Calibration was performed using a standard phantom with known dimensions, and segmentation accuracy was assessed based on inter-scan reproducibility.

### 2.4 Patient clinical data and follow-up

Follow-up was carried out at 6 and 12 months through outpatient visits and review of electronic medical records. The primary endpoint was the incidence of MACEs, defined as a composite of cardiac death, target-vessel myocardial infarction, or ischemia-driven revascularization. Secondary endpoints included all-cause hospital readmissions and bleeding events classified as Bleeding Academic Research Consortium type ≥2 (14). All clinical events were independently adjudicated by two cardiologists who were blinded to the OCT findings. Complete follow-up data were available for all patients, with no losses reported. The antiplatelet agent prescribed (clopidogrel or ticagrelor) was recorded at baseline and included as a covariate in all multivariable regression models assessing predictors of strut coverage and MACEs.

### 2.5 Environmental cold exposure assessment

Cold exposure was assessed with the CEDQ, a self-developed instrument that captures outdoor exposure, indoor heating adequacy, and protective behaviors during periods of cold weather. Each item was rated on a 4-point Likert scale (0–3), producing a cumulative score from 0 to 36.

For analytical purposes, a composite cold exposure metric that integrates three components was developed: (1) daily outdoor exposure duration (with individual questionnaire items capped at 8 h to reflect realistic limits for rural and older populations; entries exceeding this threshold were verified by follow-up telephone calls), (2) periods of inadequate indoor heating (<16 °C), and (3) time spent without adequate protective measures during cold weather. High cold exposure was defined as ≥12 h/day of total cold stress accumulated across all three domains, rather than outdoor time alone. This metric was designed to capture cumulative environmental cold burden rather than any single exposure component.

The previously described protocol demonstrated robust psychometric performance, with a content validity index of 0.92 and a test–retest intraclass correlation coefficient (ICC) of 0.91 (*P* < 0.001), as well as strong convergent validity with meteorological data (r = 0.82, *P* < 0.01).

In a subset of participants (*n* = 34), wearable temperature sensors (iButton DS1922l) recorded skin-proximal temperatures at 10-min intervals over 7 days. Participants spanning a range of age groups, occupations, and housing conditions showed a high level of agreement between CEDQ entries and sensor readings (r = 0.89, *P* < 0.001), with a bias of 0.7 h/day (limits of agreement: −2.1 to +3.5), supporting the criterion validity of the CEDQ.

Multivariable models assessing the impact of cold exposure on vascular healing were adjusted for two additional covariates: self-reported indoor temperature and a deprivation index derived from postcode-based estimates of housing insulation and household income. A threshold of ≤–20  °C was applied, consistent with the 2022 China Climate Bulletin (15).

### 2.6 Statistical analysis

Data normality was assessed with the Shapiro–Wilk test. Continuous variables are reported as mean ± standard deviation or median (IQR), as appropriate. For comparisons among three groups, normally distributed variables were analyzed using one-way ANOVA with Bonferroni correction, whereas non-normally distributed data were analyzed with the Kruskal–Wallis test followed by Dunn's *post hoc* analysis. Categorical variables are expressed as counts (%) and compared using the Chi-square test or Fisher's exact test. For two-group comparisons, the Student's *t*-test or the Mann–Whitney *U*-test was applied, depending on data distribution.

Multivariable logistic regression was performed to identify independent predictors of delayed vascular healing and 1-year MACE, with adjustment for age, sex, comorbidities, and stent characteristics. Variables with *P* < 0.1 in the univariate analysis were entered into the final multivariable model.

Longitudinal changes in NIH were assessed with linear mixed-effects models that included fixed effects for age group, time point, and their interaction, along with random subject intercepts. Inter-observer agreement for NIH measurements and strut coverage was excellent (ICC = 0.92; Cohen's *κ* = 0.85, *P* < 0.001).

Time-to-event outcomes were analyzed with Cox proportional-hazards models, adjusted for stent length, duration of diabetes, and serum 25-hydroxyvitamin D [25(OH)D] levels.

To enhance individualized risk stratification after SES implantation—particularly in older patients exposed to environmental cold—we developed a composite prediction tool, the 4D Risk Score.

The model integrates four validated predictors of MACEs: (1) uncovered-strut rate ≥10%, (2) neointimal growth rate ≥2.0 μm/month; (3) neointimal heterogeneity (IQR) ≥100 μm, and (4) prolonged cold exposure ≥72 h at ≤–20  °C (assessed with the CEDQ). Each parameter contributes one point to the total score (range, 0–4). The model was internally validated by receiver-operating-characteristic (ROC) analysis and decision-curve analysis. Risk categories were defined as low (score 0–1), moderate (2–3), and high (4) to guide post-PCI clinical management.

All statistical analyses were performed with SPSS (version 28.0) and R (version 4.2.1); statistical significance was set at *P* < 0.05.

### 2.7 Ethical statement

This study was approved by the Ethics Committee of Harbin Medical University. For the cohort of patients who underwent OCT examinations, anonymized retrospective data were used, and therefore, a waiver of informed consent was granted. In contrast, for patients who participated in the cold exposure survey, informed consent was obtained prior to participation. All data were handled in accordance with ethical guidelines, and patient confidentiality was strictly maintained throughout the study.

## 3 Results

### 3.1 Baseline characteristics and age-environment interactions

In this cohort, 71% of patients received second-generation DES. The baseline demographic, clinical, and environmental characteristics stratified by age group are summarized in Table 1. Elderly patients (> 65 years) experienced significantly longer daily cold exposure (11.5 ± 4.1 h/day; *P* < 0.001) and reported lower indoor heating temperatures (20.3 ± 1.8  °C; *P* < 0.001) than their younger counterparts. They also had higher fasting glucose concentrations (6.3 ± 1.4 mmol/L; *P* = 0.002), increased low-density lipoprotein cholesterol levels (2.9 ± 0.8 mmol/L; *P* = 0.019), and lower serum 25(OH)D concentrations (24.6 ± 6.2 ng/ml; *P* < 0.001). Collectively, these findings indicate that elderly patients face both metabolic dysregulation and environmental cold stress, which may synergistically impair vascular healing after DES implantation.

**Table 1:** Age-Stratified baseline characteristics and cold exposure metrics.

Variable	≤55 years (*n* = 46)	56–65 years (*n* = 39)	>65 years (*n* = 34)	Overall *P*-value	Pairwise comparisons
Male, *n* (%)	30 (65.2%)	22 (56.4%)	19 (55.9%)	0.008*	P1 vs. P2: *P* = 0.011, P1 vs. P3: *P* = 0.003
Hypertension, *n* (%)	21 (45.7%)	33 (84.6%)	29 (85.3%)	<0.001**	P1 vs. P2: *P* < 0.001, P1 vs. P3: *P* < 0.001
Fasting glucose (mmol/L)	5.2 ± 0.9	5.8 ± 1.1	6.3 ± 1.4	0.002*	P1 vs. P3: *P* < 0.001
LDL-C (mmol/L)	2.4 ± 0.7	2.6 ± 0.6	2.9 ± 0.8	0.019*	P1 vs. P3: *P* = 0.005
Cold exposure (hours/day, **≤**–20 °C)	7.8 ± 2.8	9.2 ± 3.3	11.5 ± 4.1	<0.001**	P1 vs. P3: *P* < 0.001, P2 vs. P3: *P* = 0.003
Indoor heating temperature ( °C)	22.5 ± 1.2	21.8 ± 1.5	20.3 ± 1.8	<0.001**	P2 vs. P3: *P* = 0.015
Serum 25(OH)D (ng/mL)	32.5 ± 8.7	28.1 ± 7.9	24.6 ± 6.2	<0.001**	P1 vs. P2: *P* = 0.007, P1 vs. P3: *P* < 0.001

P1: ≤55 years; P2: 56–65 years; P3: >65 years.

LDL-C, low-density lipoprotein cholesterol; 25(OH)D, 25-hydroxyvitamin D.

* *P*<0.05 (significant difference); **P<0.001 (highly significant difference), both based on Bonferroni-corrected threshold. This indicates the observed inter-group differences are unlikely to be caused by random chance, with higher reliability for **.

### 3.2 OCT findings: temporal and spatial healing patterns

To evaluate age-related effects on vascular healing, serial OCT assessments were performed at 6 and 12 months. At the 6-month follow-up, younger patients (≤55 years) exhibited greater neointimal formation (median NIH thickness: 90 μm, IQR 60–130) and higher strut coverage (72.1%) than elderly patients (median NIH thickness: 60 μm, IQR 50–90; strut coverage: 55.0%; both *P* < 0.001). The elderly cohort also showed the highest proportion of uncovered struts (11.7%), suggesting impaired early endothelialization.

By 12 months, elderly patients displayed a distinct change in healing trajectory, characterized by accelerated neointimal growth (3.33 μm/month vs. 0.83 μm/month; *P* < 0.001) and greater variability in NIH thickness compared with younger patients, indicating a biphasic and disorganized repair pattern.

**Figure 2:**
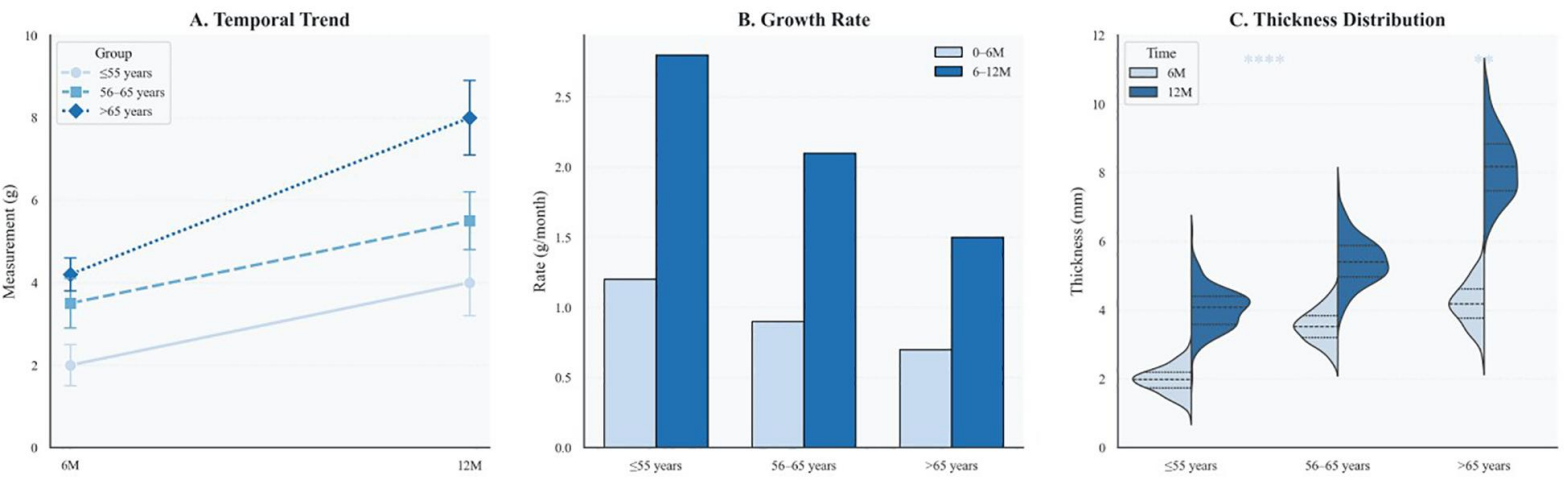
Age-specific dynamics of neointimal thickness and distribution over time. **(A)** Line plot of median neointimal thickness at 6 and 12 months across three age groups, with interquartile ranges shown as error bars. **(B)** Bar graph comparing neointimal growth rates during early (0–6 months) and late (6–12 months) phases. Color coding corresponds to time intervals and matches those in Panel C. **(C)** Violin plots display the distribution of neointimal thickness by age group and follow-up time, with quartiles indicated. Statistical significance is annotated (*P < 0.01; **P < 0.0001).

**Figure 3:**
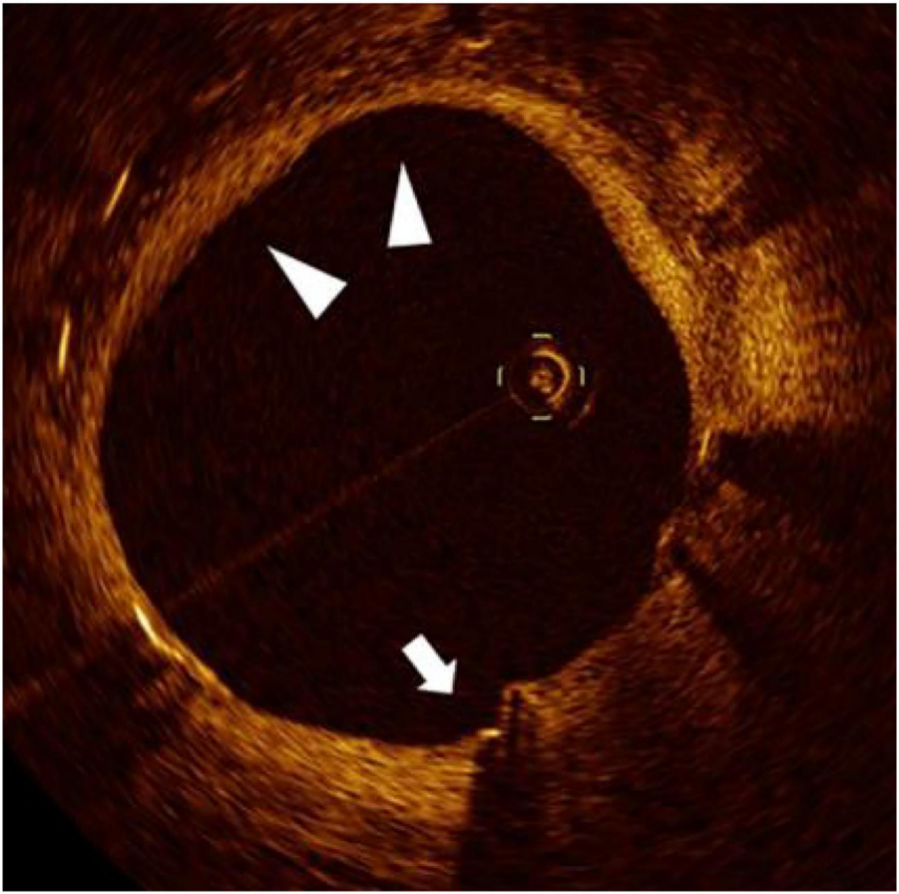
Biphasic vascular healing pattern in cold-exposed elderly patient. Representative OCT image from a 71-year-old patient with high cold exposure (CEDQ score 28, >12 h/day at ≤–20  °C) at 12 months post-stent implantation, demonstrating a characteristic biphasic healing response. White arrows indicate persistent uncovered struts, while triangles mark areas of excessive neointimal proliferation within the same cross-section. This paradoxical coexistence of under-healing and over-healing exemplifies the dysregulated vascular repair induced by the synergistic effects of aging and cold exposure.

**Figure 4:**
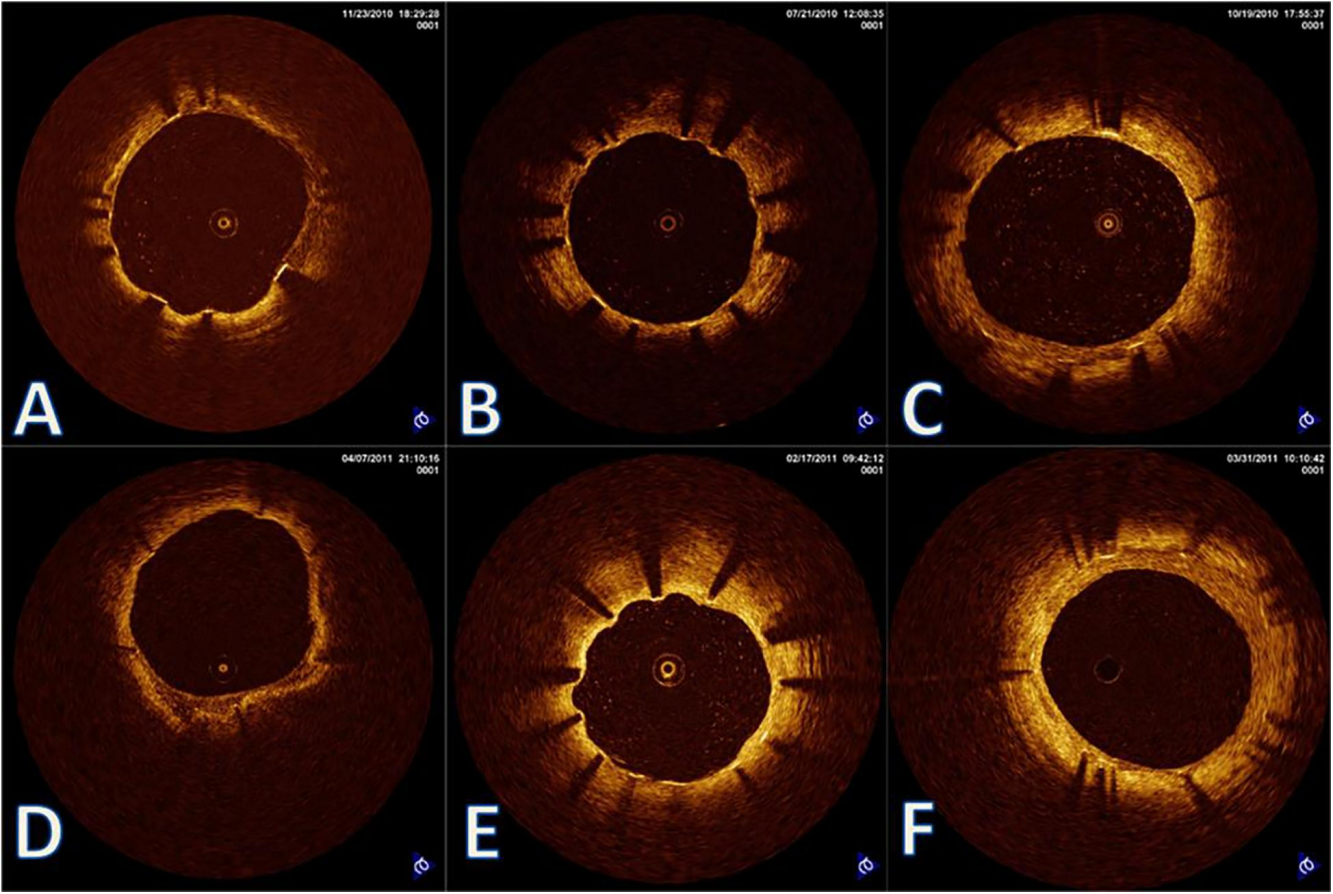
Heterogeneous vascular healing trajectories in cold-exposed elderly patients. Representative OCT images from three elderly patients (>65 years) with similar cold exposure levels showing diverse healing outcomes. **(A–C)** 6-month follow-up; Panels D-F: 12-month follow-up. **(A,D)** Patient 1: Delayed but eventually adequate healing with thin neointimal coverage at 12 months; **(B,E)** patient 2: Persistent healing failure with uncovered struts at 12 months (arrows in panel E); **(C,F)** patient 3: Accelerated healing with complete coverage but excessive neointimal proliferation at 12 months. Despite comparable age and cold exposure, these patients demonstrate the unpredictable nature of vascular healing under environmental stress, highlighting the importance of individualized risk assessment.

Age-stratified healing patterns are summarized in Table 2. Patients older than 65 years exhibited significantly delayed endothelialization at 6 months and accelerated NIH progression compared to younger groups (both *P* < 0.001). Median NIH thickness increased over time in all groups, with the elderly displaying a late-phase catch-up (Figure 2A). The elderly cohort also showed a significantly higher monthly growth rate (Figure 2B). In addition, Figure 2C demonstrates wider IQRs in the elderly, indicating greater neointimal heterogeneity during the late healing phase. Collectively, these findings suggest that advanced age is associated with biphasic, spatially heterogeneous vascular repair after stent implantation. Figure 3 illustrates the hallmark biphasic healing pattern in an elderly patient exposed to high levels of cold, showing simultaneous under- and over-healing within a single vessel segment. Figure 4 demonstrates the temporal evolution of three elderly patients with similar cold exposure, revealing pronounced heterogeneity in healing trajectories despite comparable baseline characteristics. The divergent patterns, including delayed coverage (panels A–D), persistent non-healing (panels B–E), and excessive proliferation (panels C–F), were differentially associated with clinical outcomes, with persistent non-healing exhibiting the highest rate of MACEs (45.5% vs. 18.2% for delayed healing, *P* = 0.023). These OCT findings provide a foundation for understanding the quantitative impact of cold exposure on clinical outcomes.

**Table 2:** Temporal changes in neointimal hyperplasia and strut coverage by Age group.

Parameter/Timepoint	≤55 years group (*n* = 46)	56–65 years group (*n* = 39)	>65 years group (*n* = 34)	Overall *P*-value	Pairwise comparisons
6-month follow-up					
Total deployed struts, *n*	7,057	6,643	5,771	–	–
Analyzable struts, *n* (%)	6,971 (98.8%)	6,567 (98.9%)	5,633 (97.6%)	0.092	–
Uncovered struts, *n* (%)	430 (6.1%)	485 (7.3%)	675 (11.7%)	<0.001**	P1 vs. P2: *P* < 0.001; P1 vs. P3: *P* < 0.001; P2 vs. P3: *P* = 0.023
Malapposed struts, *n* (%)	86 (1.2%)	76 (1.1%)	138 (2.4%)	<0.001**	P1 vs. P3: *P* = 0.002; P2 vs. P3: *P* = 0.001
Embedded struts, *n* (%)	5,085 (72.1%)	3,785 (57.0%)	3,176 (55.0%)	<0.001**	P1 vs. P2: *P* < 0.001; P1 vs. P3: *P* < 0.001
NIH thickness (μm), median (IQR) – 6 months	90 (60–130)	60 (40–80)	60 (50–90)	<0.001**	P1 vs. P2: *P* < 0.001; P1 vs. P3: *P* < 0.001; P2 vs. P3: *P* = 0.023
12-month follow-up					
Total deployed struts, *n*	7,377	6,412	4,610	–	–
Analyzable struts, *n* (%)	7,281 (98.7%)	6,285 (98.0%)	4,557 (98.9%)	0.156	–
Uncovered struts, *n* (%)	288 (3.9%)	212 (3.3%)	226 (4.9%)	<0.001**	P1 vs. P3: *P* < 0.001; P2 vs. P3: *P* < 0.001
Embedded struts, *n* (%)	5,629 (76.3%)	4,112 (64.1%)	3,295 (71.5%)	<0.001**	P1 vs. P2: *P* < 0.001; P1 vs. P3: *P* = 0.029
NIH thickness (μm), median (IQR) – 12 months	100 (60–150)	70 (50–110)	80 (60–110)	<0.001**	P1 vs. P2: *P* < 0.001; P1 vs. P3: *P* < 0.001; P2 vs. P3: *P* < 0.001
Monthly NIH growth rate (μm/month)	+0.83	+1.67	+3.33	<0.001**	P1 vs. P3: *P* < 0.001

IQR, interquartile range; NIH, neointimal hyperplasia.

P1: ≤55 years; P2: 56–65 years; P3: >65 years.

Strut analysis excludes struts at major side branches (diameter ≥2 mm).

Analyzable struts = total struts minus those with imaging artifacts or at excluded locations.

Monthly growth rate calculated as (12-month thickness - 6-month thickness) ÷ 6 months.

* *P* < 0.05, **P < 0.01 (Bonferroni-corrected).

### 3.3 Cold exposure and risk of MACEs: dose-response relationship

Building on the heterogeneous healing patterns observed in elderly patients, we systematically evaluated the dose-response relationship between cold exposure and clinical outcomes. Among the 119 participants, cumulative cold exposure during a 7-day period ranged from 28.0 to 64.5 h, with a median of 49.5 h. Individuals exposed to ≥56 h of ambient cold had a significantly higher incidence of MACEs than those with lower exposure (38.1% vs. 15.5%, *P* = 0.02). A positive association was observed between CEDQ score and MACE risk. In unadjusted logistic-regression analysis, each 1-point increase in CEDQ score was associated with a 24% rise in the odds of MACEs [odds ratio (OR): 1.24, 95% confidence interval (CI): 1.11–1.38, *P* < 0.001]. This relationship remained significant after adjustment for age, sex, comorbidities, indoor temperature, and the residential deprivation index (adjusted OR: 1.21, 95% CI: 1.08–1.35, *P* < 0.001).

**Figure 5:**
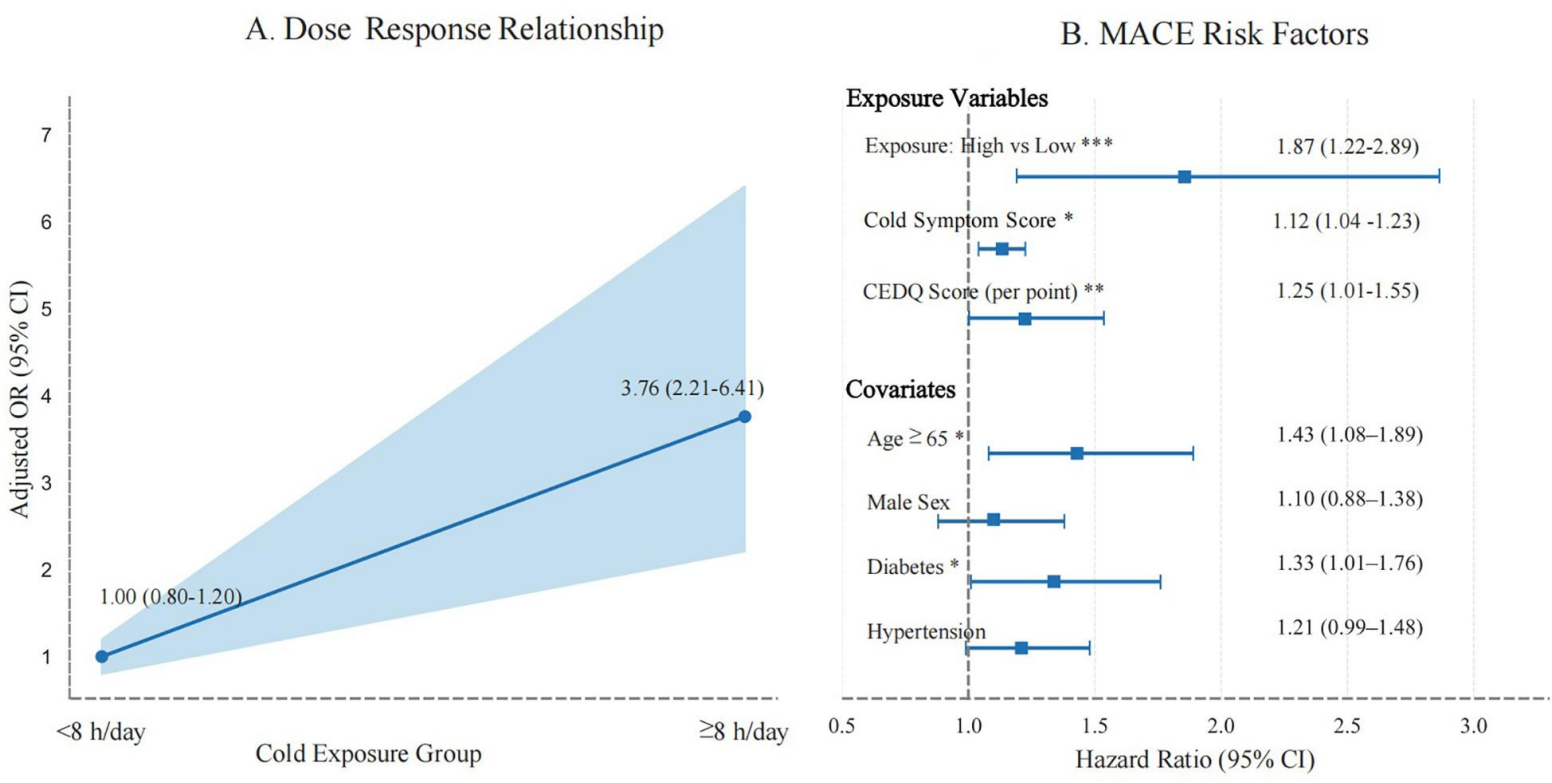
Dose-response relationship and risk factors for MACEs. **(A)** Dose-response curve showing the relationship between cumulative cold exposure (≤–20 °C) and MACEs (*P* = 0.012). **(B)** Forest plot of multivariable-adjusted hazard ratio (HR). High cold exposure (>12 h/day) is associated with a significantly increased risk of MACEs (HR: 3.42, 95% CI: 1.68–6.97). The dashed line indicates HR = 1.

Restricted cubic-spline modeling at an ambient temperature of 20 °C demonstrated a near-linear dose–response relationship, whereby each additional hour of exposure to ambient temperatures of 20 °C was associated with a 12% increase in MACE risk (*P* = 0.012) (Figure 5A). A forest plot of the multivariable-adjusted hazard ratios showed that high daily exposure to cold (≥ 12 h/day, corresponding to the ≥56 h/week threshold) was linked to a markedly increased risk of MACEs (hazard ratio 3.42, 95% CI: 1.68–6.97, *P* = 0.001) (Figure 5B). These findings further support a dose-dependent relationship between cold exposure and the occurrence of MACEs.

Table 3 summarizes the full statistical results from the two models. Model A treated the CEDQ score as a continuous variable, whereas Model B categorized cold exposure with a threshold of 56 h week. Participants in the highest exposure group (≥56 h week) had significantly greater odds of MACEs than those in the lowest exposure group (<42 h week; adjusted OR: 2.87, 95% CI: 1.32–6.25, *P* = 0.008). Both models showed significant associations between cold exposure and MACE risk, supporting the predictive validity of both continuous and categorical cold-exposure metrics.

**Table 3:** Dose-Dependent association between cold exposure and MACEs.

Variable	Model A: CEDQ score		Model B: high exposure (≥56 h/week)	
Cold exposure (main variable)	1.21 (1.08–1.35)	*P* < 0.001	2.87 (1.32–6.25)	*P* = 0.008
Age ≥65 years	1.42 (0.74–2.73)	*P* = 0.28	1.38 (0.72–2.64)	*P* = 0.31
Male sex	1.58 (0.83–3.02)	*P* = 0.17	1.61 (0.85–3.08)	*P* = 0.14
Comorbidity index (per point)	1.35 (1.10–1.66)	*P* = 0.003	1.34 (1.09–1.65)	*P* = 0.005
Indoor temperature <16 °C	1.93 (1.05–3.53)	*P* = 0.033	1.85 (1.01–3.41)	*P* = 0.039
Deprivation index (per SD increase)	1.41 (1.11–1.79)	*P* = 0.004	1.39 (1.10–1.76)	*P* = 0.006

MACEs, major adverse cardiovascular events; CEDQ, cold exposure diary questionnaire; SD, standard deviation.

### 3.4 Predictive value of spatial heterogeneity and the 4d risk score

**Figure 6:**
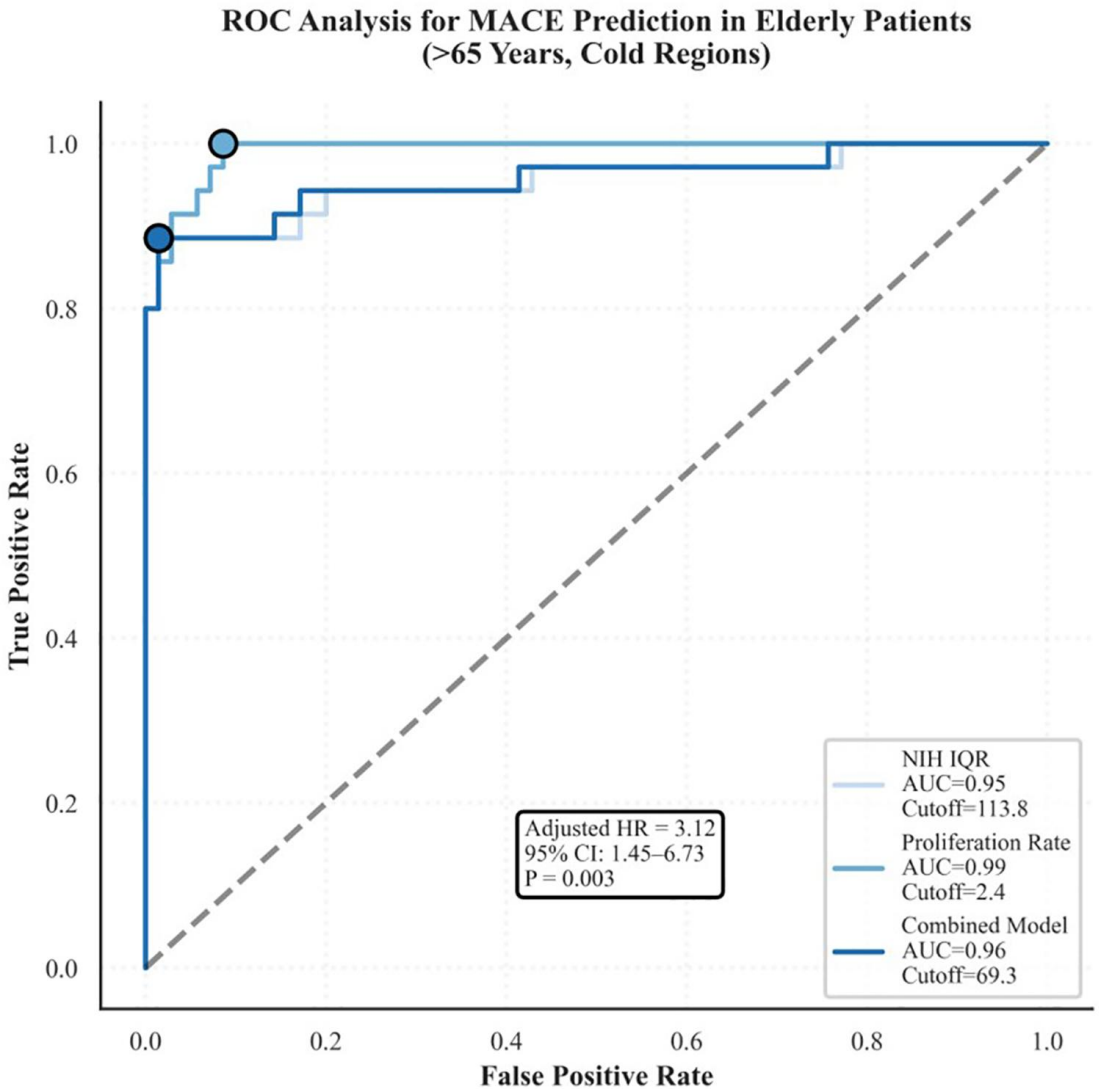
Kaplan–Meier survival curves for patients with high or low spatial heterogeneity. High heterogeneity (IQR >120 μm) was associated with significantly lower MACE-free survival (*P* < 0.001), with an AUC of 0.72, indicating moderate predictive accuracy.

Spatial heterogeneity of NIH, defined as an IQR >120 μm, emerged as an independent predictor of MACEs (16). ROC analysis demonstrated moderate discriminative performance, with an area under the curve (AUC) of 0.72. Kaplan–Meier survival analysis confirmed that patients with pronounced spatial heterogeneity had significantly lower MACE-free survival (*P* < 0.001) (Figure 6).

In the composite 4D Risk Score (Table 4), all four parameters (i.e., uncovered strut rate, neointimal growth rate, spatial heterogeneity, and cold exposure) were independently associated with MACEs, yielding hazard ratios of 2.91, 3.12, 3.42, and 3.25, respectively. The model displayed excellent performance, with an internally validated AUC of 0.83.

**Table 4:** Multivariable Cox proportional hazards model for cold exposure-related MACE risk.

Exposure type & variable	Adjusted HR	95% CI	*P*-value
Categorical modeling			
Moderate exposure (8–12 h/day)	1.65	0.98–2.78	0.058
High exposure (>12 h/day)	3.42	1.68–6.97	0.01**
Continuous exposure modeling			
Cold exposure (per 1 h/day increase)	1.12	1.01–1.24	0.012*
Covariates			
Age (per year increase)	1.04	1.01–1.08	0.015*
Male sex	1.12	0.65–1.92	0.670
Hypertension	1.34	0.80–2.22	0.270
Diabetes mellitus	1.45	0.85–2.46	0.175
LDL-C (per 1 mmol/L increase)	1.20	1.02–1.42	0.031*
DAPT duration (per month increase)	0.96	0.91–1.01	0.110

MACE, major adverse cardiovascular event; LDL-C, low-density lipoprotein cholesterol; DAPT, dual antiplatelet therapy.

* *P* < 0.05, **P < 0.01 (Bonferroni-corrected if applicable).

**Table 5:** 4D risk score criteria and recommended risk-stratified management.

Parameter	Threshold	Score	Management recommendation
Uncovered struts	≥10% at 6 months (by OCT)	1	Consider extended DAPT
Neointimal proliferation rate	>2.0 μm/month at 12 months	1	Intensify OCT follow-up every 3 months
Neointimal heterogeneity (IQR)	>100 μm	1	Monitor for delayed healing via serial OCT
Cold exposure (environmental)	≥72 h at ≤–20 °C (assessed by CEDQ)	1	Advise lifestyle changes to reduce exposure
Total score (0–4)	0–1: Low risk (routine care); 2–3: Moderate risk (enhanced OCT); 4: High risk (intensive follow-up + DAPT adjustment)		

CEDQ, cold exposure diary questionnaire; DAPT, dual antiplatelet therapy; OCT, optical coherence tomography; IQR, interquartile range.

**Figure 7:**
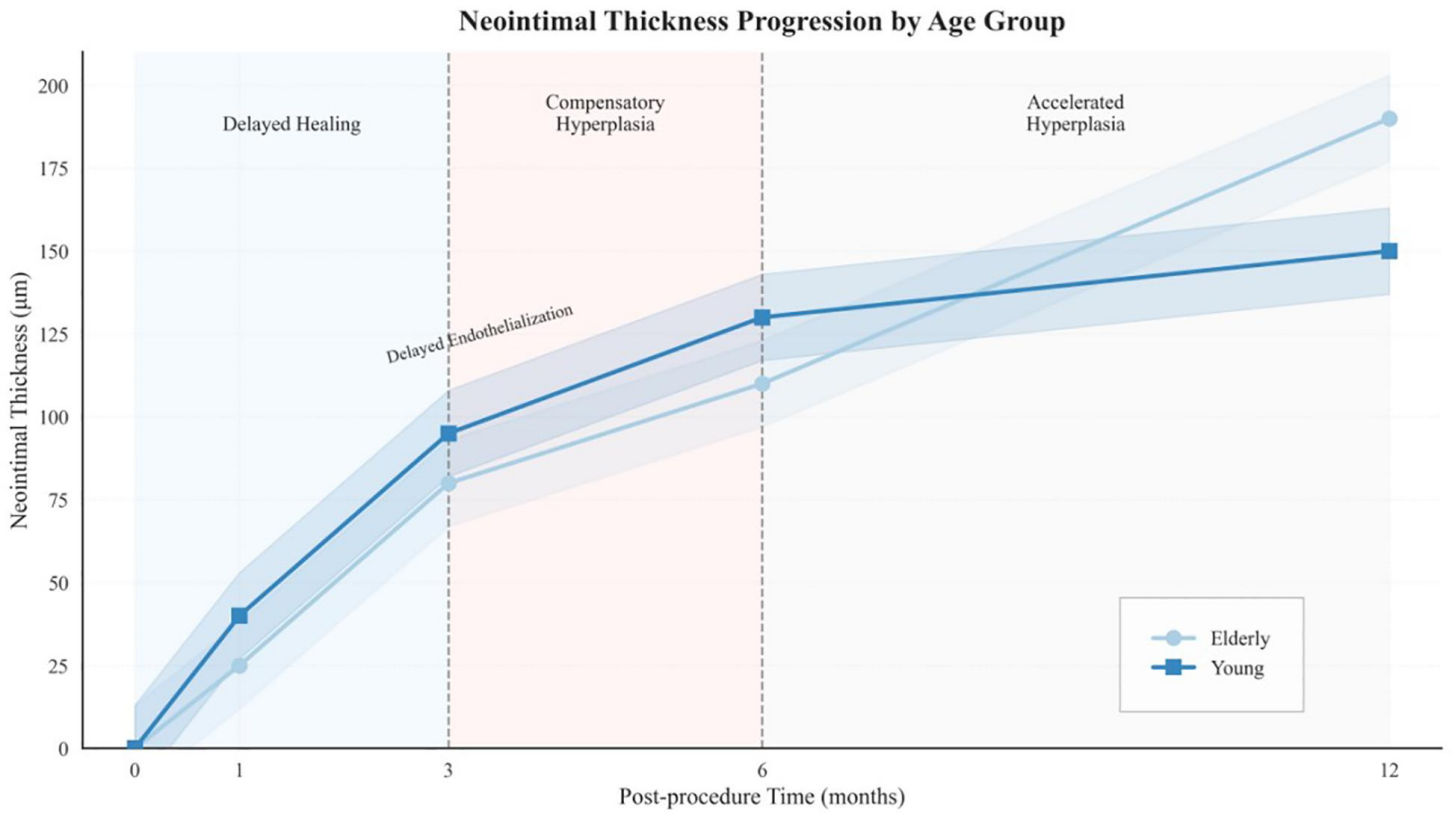
Conceptual model of biphasic healing dynamics in elderly patients. Illustration of a biphasic vascular healing pattern observed in elderly patients. Phase I (0–6 months) is characterized by delayed endothelialization and reduced neointimal formation, whereas Phase II (6–12 months) is marked by exaggerated neointimal proliferation. This pattern reflects the interplay between biological aging and cold-induced vascular stress.

**Figure 8:**
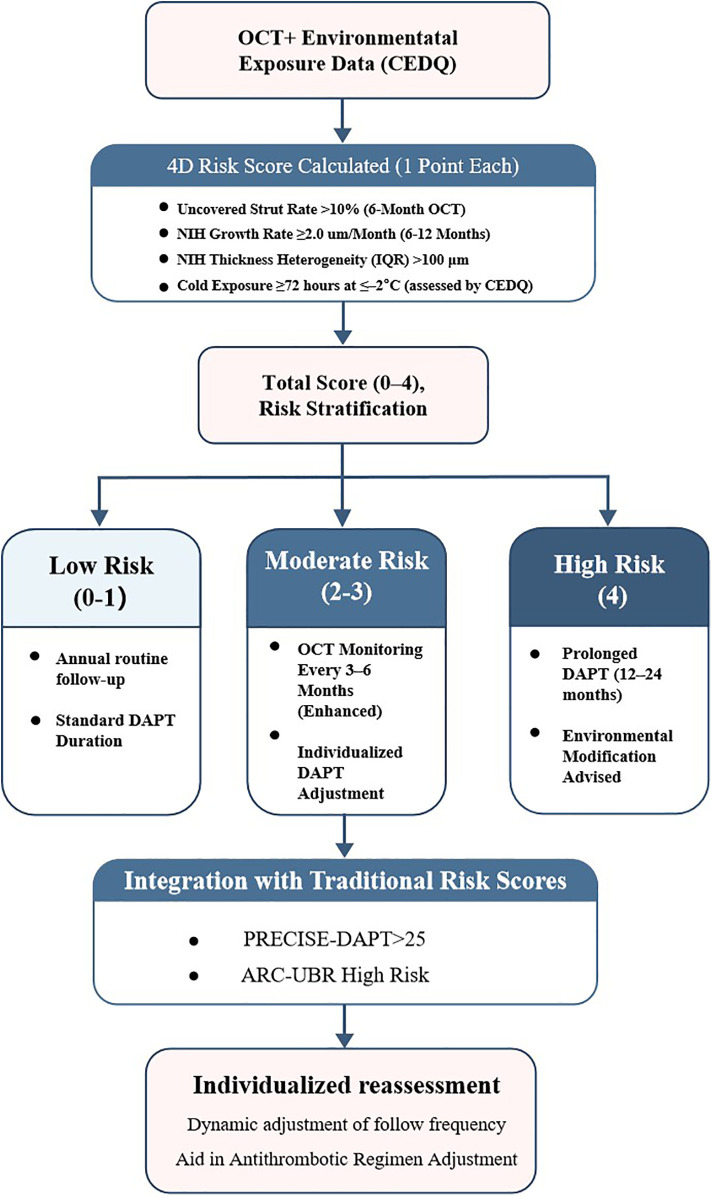
Proposed 4D risk-based management algorithm following DES implantation.

A stepwise algorithm illustrating how the 4D Risk Score can inform management after DES implantation is provided in Figure 7. In ROC analysis of the test dataset, the AUC was 0.63 (Figure 8A), indicating only moderate discrimination. This value is lower than initially reported, likely reflecting optimism inherent to internal validation. Nevertheless, decision-curve analysis (Figure 8B) demonstrated a consistent net clinical benefit across a wide range of threshold probabilities, outperforming both “treat-all” and “treat-none” strategies and underscoring the clinical utility of the score in optimizing therapeutic decisions. The proposed 4D Risk Score helps in personalized risk assessment and management strategies after PCI. The detailed criteria for scoring and management recommendations are outlined in Table 5.

Collectively, these findings establish the 4D Risk Score as a practical and clinically meaningful tool for individualized risk stratification after PCI, particularly in older patients exposed to environmental cold stress.

## 4 Discussion

### 4.1 Biphasic vascular healing pattern

**Figure 9:**
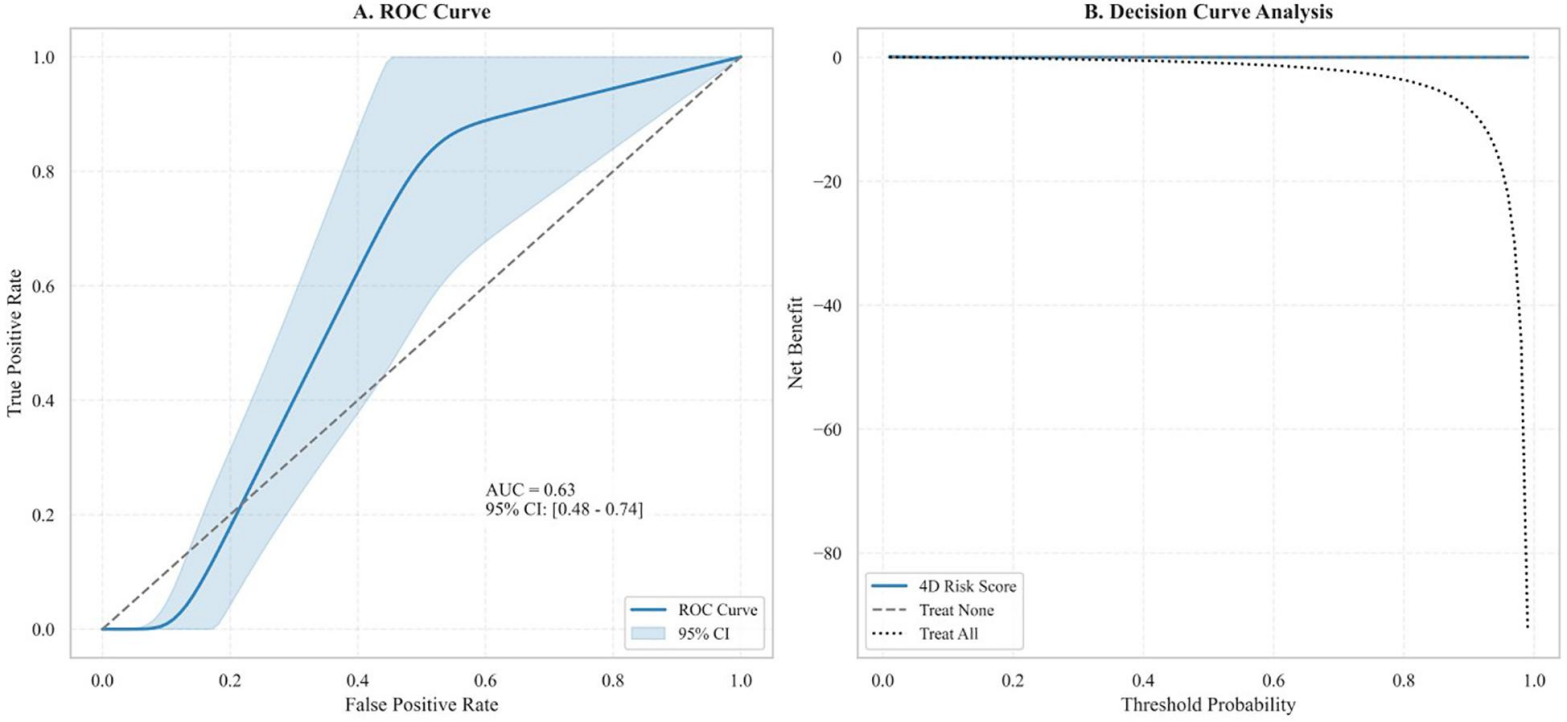
ROC curve analysis and decision curve analysis of the 4D risk score. **(A)** ROC curve demonstrates the model's discriminatory ability for predicting MACEs (AUC: 0.63, 95% CI: 0.48–0.74; *P* = 0.041). The shaded region delineates the 95% CI. **(B)** Decision curve analysis shows the net clinical benefit of the 4D Risk Score across varying threshold probabilities. Compared to the “Treat All” and “Treat None” strategies, the model yielded marginal net benefit, primarily at lower thresholds, indicating limited incremental utility in clinical decision-making.

This study identifies a biphasic vascular-healing trajectory in elderly patients after SES implantation in cold environments. This pattern, conceptually illustrated in Figure 9, consists of two distinct phases: In the early phase (0–6 months), healing was markedly delayed, as reflected by higher proportions of uncovered struts and thinner neointimal coverage than in younger adults. During the late phase (6–12 months), neointimal growth accelerated and spatial heterogeneity increased, indicating a compensatory yet dysregulated repair response. Importantly, a dose–response relationship was evident: prolonged cold exposure was associated with a higher uncovered-strut rate and greater neointimal proliferation. These findings suggest that cold exposure functions as a cumulative vascular stressor that amplifies age-related impairments in endothelial recovery.

The delay in early healing may be driven by cold- and age-induced disruptions of vascular homeostasis. Acute cold exposure precipitates sympathetic over-activation and vasoconstriction by elevating circulating norepinephrine and stimulating *α*-adrenergic receptors, thereby impairing endothelial reactivity. Chronic cold stress, meanwhile, promotes the release of pro-inflammatory cytokines (e.g., interleukin-6 and tumor necrosis factor-α), leading to delayed endothelial regeneration and destabilized neointimal scaffolding. In addition, mitochondrial dysfunction in endothelial progenitor cells (EPCs) restricts β1-integrin-mediated migration (17, 18), further contributing to delayed endothelialization.

Aging further exacerbates this dysfunction through telomere attrition, which activates the ataxia-telangiectasia mutated/nuclear serine/threonine protein kinase Chk2/p53 pathway in vascular smooth muscle cells (VSMCs), leading to G1 cell cycle arrest and a 42% reduction in proliferative capacity compared to younger individuals (19). As a result, neointimal coverage becomes thinner and displays greater spatial heterogeneity (median thickness: 60 μm vs. 90 μm; a 75% increase in IQR).

During the chronic phase (6–12 months) of healing, telomere-dysfunction-driven stress responses sustain inflammation and epigenetic drift. Up-regulation of the long non-coding RNA TERRA triggers Toll-like receptor-9/nuclear factor *κ*B signaling, enhancing monocyte recruitment and extracellular-matrix remodeling. Concomitantly, hypermethylation of the Krüppel-like factor**-**4 promoter (*β* > 0.8) induces phenotypic switching in VSMCs, evidenced by an 80% reduction in the contractile marker α-smooth-muscle actin and a 320% increase in the osteogenic marker osteopontin (20, 21). This phenotypic plasticity drives NIH and metabolic reprogramming via glycolytic enzymes (e.g., 6-phosphofructo-2-kinase/fructose-2,6-bisphosphatase-3), increasing cellular-proliferation heterogeneity (IQR 1.7 → 4.3) (22). However, these pathways remain hypothetical because molecular assays (e.g., methylation profiling, RNA sequencing) were not performed. Multivariable analysis in this study identified aging as an independent predictor of delayed vascular healing, highlighting the need for age-adapted OCT thresholds.

Our results align with those of Tomaniak et al. (23), who reported delayed neointimal coverage in elderly patients at 9 months. Unlike single-time-point studies, we propose a temporal healing trajectory comprising an initial phase of EPC-related dysfunction followed by inflammation-driven HIN. This biphasic pattern, supported by serial OCT data and spatial-heterogeneity metrics, reflects the interplay between aging and cold-induced vascular stress.

### 4.2 Environmental and aging interactions and risk stratification

Biological aging and chronic cold exposure exert a synergistic “dual-hit” effect on vascular healing, delaying re-endothelialization and increasing the risk of MACEs. Cold stress promotes oxidative damage and accelerates telomere attrition—two hallmarks of vascular senescence—thereby impairing smooth-muscle repair and endothelial regeneration. In north-eastern China, extreme winter conditions intensify vascular vulnerability through two primary mechanisms:

#### 4.2.1 Cold-induced endothelial dysfunction

This dysfunction correlates with CEDQ scores, although mechanistic validation is pending. Wearable thermometric telemetry or flow-mediated dilation testing could provide real-time insights into endothelial function under cold stress.

#### 4.2.2 TRPM8 activation in endothelial and smooth muscle cells (hypothesis)

In endothelial cells, existing literature suggests that TRPM8 activation inhibits vascular endothelial growth factor–mediated angiogenesis by 38% via the Ca^2^^+^/calcineurin–nuclear factor of activated T cells signaling (24, 25). In VSMCs, TRPM8 activation is proposed to enhance ERK1/2 and cyclin-D1 activity, thereby promoting late-phase neointimal growth. These mechanisms are supported by previous studies; however, molecular measurements were not performed in our study and are required for further validation (26).

#### 4.2.3 Air pollution as a compounding stressor

Severe ambient pollution (PM2.5 >150 μg/m^3^) further aggravates cold-induced vascular stress through oxidative-injury pathways, producing a 2.3-fold increase in MACE risk relative to temperate regions (*P* = 0.012) (27). In elderly patients, conventional OCT-derived neointimal-thickness thresholds (e.g., ≥40 μm) may underestimate healing deficits because focal hyperplasia can mask adjacent uncovered struts, giving false reassurance regarding vascular repair (28, 29). In our cohort, OCT-defined strut coverage correlated only moderately with functional endothelial recovery (r = 0.62, *P* = 0.03), highlighting the limitations of OCT alone. Consequently, multimodal assessments that integrate functional imaging (e.g., fractional flow reserve), inflammatory biomarkers, and quantitative cold-exposure metrics (e.g., CEDQ scores, wearable-sensor data) are warranted (2).

### 4.3 Limitations

This study has several limitations. First, its retrospective, non-randomized design restricts causal inference, particularly regarding the effect of cold exposure on vascular healing. Second, the choice of antiplatelet therapy (ticagrelor vs. clopidogrel) was not standardized and may have influenced endothelial recovery through distinct platelet–vascular interactions (30). Third, although OCT provides high-resolution structural information, it does not capture vascular function (e.g., shear stress, nitric-oxide bioavailability, or endothelial nitric-oxide synthase activity) (31); multimodal imaging (such as fractional flow reserve or near-infrared spectroscopy) should therefore be considered. Fourth, the 12-month follow-up may have missed very-late events, including stent thrombosis or chronic restenosis. Longer-term studies are needed to assess sustained vascular responses to aging and cold exposure. Fifth, although most patients received second-generation DESs (71%), the findings may not be generalizable to third-generation stents or bioresorbable scaffolds, which could differ in healing kinetics under environmental stress (32). Sixth, the elderly subgroup was relatively small (*n* = 34), limiting the power to detect sex-based differences or gene–environment interactions (e.g., TRPM8 polymorphisms or oestrogen-related endothelial effects) (33). In addition, the absence of physiological measurements (e.g., flow-mediated dilation, circulating endothelial microparticles) limits mechanistic interpretation beyond OCT-derived metrics. Future studies should incorporate such biomarkers to clarify healing heterogeneity. Lastly, while cold-induced vasodilation is known to provide insights into vascular health, this study did not employ non-invasive methods such as diffuse speckle pulsatile flowmetry to assess it. Given its potential as a non-invasive technique to study endothelial function under cold stress, incorporating such methods in future studies could provide valuable additional data and help clarify the mechanisms of vascular healing in response to cold exposure.

## 5 Conclusion

In elderly patients living in cold climates, a biphasic vascular**-**healing response after SES implantation was observed: re-endothelialization was delayed during the early phase, whereas neointimal proliferation was exaggerated during the late phase. High-resolution OCT revealed substantial spatial heterogeneity, particularly under prolonged cold exposure. These findings indicate a synergistic impact of aging and environmental cold on vascular repair and highlight the need for climate-aware post-PCI surveillance. Preliminary risk modelling that integrates environmental and biological markers showed predictive potential for MACEs, although broader validation is required. Given the study's retrospective design and the small elderly subgroup, the results should be interpreted with caution. Prospective, multicenter studies are warranted to confirm these observations and to inform individualized strategies for vulnerable populations exposed to extreme environmental stress.

## Data Availability

The original contributions presented in the study are included in the article/Supplementary Material, further inquiries can be directed to the corresponding author.
