# Supplementary material for: Cold-driven biphasic vascular healing in elderly patients: 4D optical coherence tomography stratification of major adverse cardiovascular event risk based on age-environment interactions
Source: Front Cardiovasc Med. 2025 Nov 13;12:1663394. doi: 10.3389/fcvm.2025.1663394 (PMC12658980; doi:10.3389/fcvm.2025.1663394)
Supplement: Supplementary file 1 [file Datasheet1.pdf]

Cold Exposure Diary Questionnaire

Version: CHES-2024v1

Section 1: Basic Information

1. Personal Information

Name: ____________________

Gender: □ Male □ Female

Date of Birth: ____ Year ____ Month ____ Day

Contact Information: ____________________

2. Age Group (Please check)

□ ≤55 years (Code 0, Correction Coefficient W_age=1.0)

□ 56-65 years (Code 1, Correction Coefficient W_age=1.2)

□ >65 years (Code 2, Correction Coefficient W_age=1.5)

3. Type of Residence (Please check)

□ Urban centralized heating (Code 0, Spatial Correction S_factor=1.0)

□ Rural self-heating (Code 1, Spatial Correction S_factor=1.3)

□ Migratory (Winter migration to warm cities, Code 2, Spatial Correction S_factor=0.7)

4. Profession Type (Please check)

□ No outdoor work (Code 0, Occupational Coefficient O_factor=0.5)

□ Intermittent outdoor work (Code 1, Occupational Coefficient O_factor=1.0)

□ Continuous outdoor work (Code 2, Occupational Coefficient O_factor=1.5)

Section 2: 24-Hour Activity Log

Please record the activities of the previous day by hour (Example is filled in)

Time Period Activity Type Micro-Environmental Characteristics Perceived Temperature Level Wind Speed Perception Protective Measures

05:00-06:00 □ Morning Exercise □ Commute □ Other______ □ Open Area □ Between Buildings □ Indoor □ Extremely Cold (-5~-3℃) □ No Perception (<3m/s) □ No Protection (1.0)

06:00-07:00 ... ...

22:00-23:00 □ Housework □ Leisure □ Other______ □ Central Heating Room □ Self-Heating Room □ Warm (18-22℃) □ No Perception □ Mild □ Obvious □ Electric Blanket □ Hot Water Bag □ None

Section 3: Environmental and Behavioral Assessment

5. Indoor Environmental Monitoring

Heating Temperature:

□ ≥22℃ (Correction Coefficient T_indoor=0.8)

□ 18-22℃ (Correction Coefficient T_indoor=1.0)

□ <18℃ (Correction Coefficient T_indoor=1.3)

Temperature Fluctuation (Indoor Day-Night Temperature Difference):

□ <3℃ (Fluctuation Coefficient ΔT=1.0)

□ 3-5℃ (Fluctuation Coefficient ΔT=1.2)

□ >5℃ (Fluctuation Coefficient ΔT=1.5)

Section 4: Physiological Response and Health Status

7. Cold-Related Symptoms (Past Week)

Symptom None (0 points) Occasional (1 point) Frequent (2 points)

Numbness of fingers lasting >30 minutes □ □ □

Morning chest pain/chest tightness □ □ □

Skin cyanosis/pallor □ □ □

Total Score: ________ (P_factor=Total Score×0.3)

Section 5: Medication Use

8. Medication Use

Beta-Blockers:

□ Yes (Current resting heart rate: ____ bpm, If <60 bpm, Correction Coefficient M=1.2)

□ No (M=1.0)

Anticoagulants (e.g., Aspirin):

□ Regularly taken (Correction Coefficient B=0.8)

□ Not taken (B=1.0)

Section 6: Special Group Assessment

9. Migratory Population Assessment

Migration Destination: □ Sanya □ Other Warm Cities: ________

Main Activity during Migration:

□ Resting at home (Correction Coefficient H=0.7)

□ Outdoor activity (H=1.2)

□ Tourism/Short-term stay (H=1.0)

Adaptation after return:

□ Recovery within 3 days (Adaptation Coefficient A=0.9)

□ More than 1 week of discomfort (A=1.3)

Section 7: Exposure Dose Calculation and Result Output

11. Total Exposure Dose Formula

$$TE_{total} = [\ \Sigma\ (\text{Activity Hours} \times T_{level} \times W_{wind} \times \text{Protective Coefficient})\ ] \times (W_{age} \times S_{factor}) / (1 + 0.1 \times PM_{2.5}) + P_{factor} + (\Delta T \times 0.5) + (M \times B \times R)$$

Parameter Explanations:

T_{level}: Extremely Cold=1.5 / Cold=1.2 / Mild Cold=1.0

W_{wind}: No Perception=1.0 / Obvious=1.3 / Blocked=1.5

PM2.5: Local real-time concentration (default $35\mu g/m^3$ if not monitored)

Section 8: Risk Level Classification and Recommendations

12. Risk Level Classification

Total Exposure Value (TE_total) Risk Level Intervention Recommendations

<15 Low Risk Regular Protection

15-30 Medium Risk Medical Monitoring + Behavioral Intervention

>30 High Risk Immediate Medical Attention + Environmental Modification

Section 9: Data Verification and Quality Control

13. Data Authenticity Verification

GPS Tracking Comparison: Allow access to mobile location data? □ Yes □ No

Temperature Calibration: If self-reported temperature differs from meteorological data by >2°C, initiate manual verification process

Section 10: Biomarker Re-Testing (For High-Risk Individuals)

14. Biomarker Re-Testing

Indicator Test Value Test Date

Serum Endothelin-1 (ET-1) ____ /

Fibrinogen ____ /
